# A pilot study of a ketogenic diet in bipolar disorder: clinical, metabolic and magnetic resonance spectroscopy findings

**DOI:** 10.1192/bjo.2024.841

**Published:** 2025-02-25

**Authors:** Iain H. Campbell, Nicole Needham, Helen Grossi, Ivana Kamenska, Saturnino Luz, Shane Sheehan, Gerard Thompson, Michael J. Thrippleton, Melissa C. Gibbs, Joana Leitao, Tessa Moses, Karl Burgess, Benjamin P. Rigby, Sharon A. Simpson, Emma McIntosh, Rachel Brown, Ben Meadowcroft, Frances Creasy, Maja Mitchell-Grigorjeva, John Norrie, Ailsa McLellan, Cheryl Fisher, Tomasz Zieliński, Giulia Gaggioni, Harry Campbell, Daniel J. Smith

**Affiliations:** Division of Psychiatry, Centre for Clinical Brain Sciences, University of Edinburgh, UK; Department of Nutrition and Dietetics, Royal Hospital for Children and Young People, NHS Lothian, Edinburgh, UK; Usher Institute, University of Edinburgh, UK; Centre for Medical Informatics, University of Edinburgh, UK; Centre for Clinical Brain Sciences, University of Edinburgh, UK; Edinburgh Imaging Facility, University of Edinburgh, UK; Centre for Engineering Biology, School of Biological Sciences, University of Edinburgh, UK; Institute of Quantitative Biology, Biochemistry and Biotechnology, University of Edinburgh, UK; Population Health Sciences Institute, Newcastle University, UK; School of Health and Wellbeing, University of Glasgow, UK; Health Economics and Health Technology Assessment, University of Glasgow, UK; Department of Psychiatry, NHS Lothian, Edinburgh, UK; Impact and Development, Bipolar Scotland, Edinburgh, UK; Department of Paediatric Neurology, Royal Hospital for Children and Young People, NHS Lothian, Glasgow, UK

**Keywords:** Bipolar disorder, ketogenic diet, ketosis, magnetic resonance spectroscopy, metabolic psychiatry

## Abstract

**Background:**

Preliminary evidence suggests that a ketogenic diet may be effective for bipolar disorder.

**Aims:**

To assess the impact of a ketogenic diet in bipolar disorder on clinical, metabolic and magnetic resonance spectroscopy outcomes.

**Method:**

Euthymic individuals with bipolar disorder (*N* = 27) were recruited to a 6- to 8-week single-arm open pilot study of a modified ketogenic diet. Clinical, metabolic and MRS measures were assessed before and after the intervention.

**Results:**

Of 27 recruited participants, 26 began and 20 completed the ketogenic diet. For participants completing the intervention, mean body weight fell by 4.2 kg (*P* < 0.001), mean body mass index fell by 1.5 kg/m^2^ (*P* < 0.001) and mean systolic blood pressure fell by 7.4 mmHg (*P* < 0.041). The euthymic participants had average baseline and follow-up assessments consistent with them being in the euthymic range with no statistically significant changes in Affective Lability Scale-18, Beck Depression Inventory and Young Mania Rating Scale. In participants providing reliable daily ecological momentary assessment data (*n* = 14), there was a positive correlation between daily ketone levels and self-rated mood (*r* = 0.21, *P* < 0.001) and energy (*r* = 0.19 *P* < 0.001), and an inverse correlation between ketone levels and both impulsivity (*r* = −0.30, *P* < 0.001) and anxiety (*r* = −0.19, *P* < 0.001). From the MRS measurements, brain glutamate plus glutamine concentration decreased by 11.6% in the anterior cingulate cortex (*P* = 0.025) and fell by 13.6% in the posterior cingulate cortex (*P* = <0.001).

**Conclusions:**

These findings suggest that a ketogenic diet may be clinically useful in bipolar disorder, for both mental health and metabolic outcomes. Replication and randomised controlled trials are now warranted.

A ketogenic diet is established as a metabolic therapy for refractory epilepsy, supported by data from 13 randomised controlled trials (RCTs) and over a century of clinical use.^1,2^ Preliminary case report, observational and pilot data have also suggested that a ketogenic diet may have beneficial effects in bipolar disorder.^3–6^ Epilepsy and bipolar disorder may share some common pathophysiological mechanisms and several antiseizure medications are useful for bipolar disorder. Research into the effects of a ketogenic diet has highlighted mechanisms that may be relevant to bipolar disorder, including neuroprotective, anti-inflammatory and positive metabolic effects.^7^ We have previously proposed a mechanism of action of a ketogenic diet that may address deficits in brain cellular energetics (impaired insulin signalling and dysregulated glucose metabolism).^8,9^

## Cardiometabolic health and bipolar disorder

Bipolar disorder carries a substantial metabolic burden, with high rates of obesity, type 2 diabetes, cardiovascular disease and reduced life expectancy.^10,11^ Several current first-line medications for bipolar disorder increase cardiometabolic risk: adjunctive treatment strategies to ameliorate these effects are urgently needed.^12^ A ketogenic diet has been demonstrated to improve cardiometabolic health outcomes within general population samples.^13^ This pilot study was conducted primarily to assess the feasibility and acceptability of a ketogenic diet intervention in euthymic patients with bipolar disorder. These primary outcomes have been reported previously.^14^ To inform the design of a future RCT, we also assessed a range of secondary clinical, metabolic and brain magnetic resonance spectroscopy (MRS) outcomes, which are the focus of this report.

## Method

This was a single-group, non-randomised open interventional pilot study, with no control group. The study was registered at isrctn.com, with the registration number ISRCTN61613198, on 2 March 2022. The authors assert that all procedures contributing to this work comply with the ethical standards of the relevant national and institutional committees on human experimentation and with the Helsinki Declaration of 1975, as revised in 2013. All procedures involving human participants/patients were approved by the South East Scotland Research Ethics Committee 02 (REC ref 22/SS/0007) and National Health Service (NHS) Lothian Research and Development. The Academic and Clinical Central Office for Research and Development (ACCORD) provided sponsorship. Written informed consent was obtained from all participants.

### Study participants

Recruitment for study participants began on 27 April 2022 and was carried out in collaboration with the charity Bipolar Scotland, via local support groups, social media and the Bipolar Scotland newsletter.

#### Participant eligibility criteria

Participants were individuals diagnosed with bipolar disorder, according to DSM-IV^15^ criteria. Eligibility was contingent upon a period of clinical euthymia lasting at least 3 months, defined by an absence of clinically significant depressive or hypomanic/manic episodes. The age range for participation was 18–70 years. Understanding of English language and residence in Scotland were also required.

#### Initial exclusion criteria

The initial exclusion criteria excluded participants with conditions including pregnancy, breastfeeding, intention to conceive within 3 months, active substance misuse, recent adherence to a ketogenic diet (within the past 2 months), adherence to a vegan diet (vegan ketogenic diet is widely used but our team did not have experience delivering this), recent hospital admission (within the past 3 months), concurrent participation in other research studies, inability to complete baseline assessments and a history of liver, kidney or cardiovascular diseases. Severe hyperlipidaemia, indicated by familial hypercholesterolaemia or a total cholesterol level exceeding 7.5 mmol/L, was also an exclusion criterion.

#### Additional exclusion criteria applied during the study

As the pilot study progressed, further exclusion criteria were added to address emergent considerations. These were a diagnosis of diabetes, engagement in activities requiring a high energy expenditure (such as long-distance running) and significant recent changes to psychotropic medication use.

### Participant assessments and data collection

#### Screening and baseline procedures

Participants were briefed about the study's objectives and the eligibility criteria were assessed. On successful screening and consent, baseline assessments were conducted and participants were provided with comprehensive guidelines on adopting and adhering to a ketogenic diet. This included information on potential risks and detailed instructions for diet monitoring. Participants were also required to submit a 3-day food diary and a pre-ketogenic diet information sheet in advance to facilitate the customisation and planning of their diet by the study dietitian.

#### Assessment and monitoring

Assessments were conducted at baseline and during the follow-up period (6–8 weeks). Baseline assessment included a review of medical and medication histories, measurements of blood pressure and body mass index (BMI) and completion of a diagnostic interview. Participants also completed several mental health symptom scales, including the Affective Lability Scale-18 (ALS-18), Beck Depression Inventory (BDI) and the Young Mania Rating Scale (YMRS). Quality of life was assessed using the Within Trial Resource Use Questionnaire, the EuroQol 5D and the Work Productivity and Activity Impairment Questionnaire, tailored to capture data on health and social care resource utilisation, household expenditure on food and beverages and information on employment and absenteeism. Fasting venepuncture and MRS brain scans were conducted pre- and post-intervention. Post-analysis of the study data it was ascertained that one participant had not fasted on the morning of their baseline appointment for venepuncture and MRS. Their data did not present as an outlier for any variable or significantly alter the findings of the study and so remains in the analysis. Data collection was facilitated through both paper questionnaires and a secure online platform, with face-to-face interactions occurring at designated clinical and imaging facilities.

#### Brain magnetic resonance

Brain magnetic resonance imaging (MRI) and spectroscopy were acquired using a 3-T clinical MRI scanner (MAGNETOM Prisma, Siemens Healthcare, Erlangen, Germany) with a 32-channel receive head coil. A three-dimensional T1-weighted image (T1w; repetition time/echo time/inversion time = 2500/4.37/1100 ms, flip angle = 7°, 1.0 mm isotropic resolution) was acquired to facilitate placement of MRS voxels covering the anterior cingulate cortex (ACC; 20 × 20 × 20 mm^3^, 128 averages, acquisition time 4 m 26 s), right dorsolateral prefrontal cortex (RDLPFC; 30 × 15 × 15 mm^3^, 96 averages, acquisition time 3 m 22 s) and posterior cingulate cortex (PCC; 20 × 20 × 20 mm^3^, 128 averages, acquisition time 4 m 26 s). In test scans and in the first study scan, we did not achieve consistently narrow linewidth and high spectral quality in the basal ganglia volume of interest (VOI). We therefore changed the VOI to the RDLPFC based on previous MRS literature indicating impaired metabolism in mood disorders in this brain region.^16^ The ACC VOI was also selected based on previous literature implicating this region in the pathophysiology of bipolar disorder,^17^ while the PCC VOI was selected to generate optimal data quality through lower *B*_0_ inhomogeneity and reduced likelihood of lipid contamination from the scalp. At follow-up, voxels were automatically placed at the same location using the Siemens’ AutoAlign feature. MRS was obtained using point-resolved spectroscopy (PRESS; repetition time/echo time = 2000/30 ms, flip angle = 90°, receive bandwidth 4000 Hz, 4096 data points, with a single transient acquired without water suppression). Please see Supplementary materials available at https://doi.org/10.1192/bjo.2024.841 for further details.

#### Daily monitoring and rest/activity pattern tracking

Participants engaged in daily self-monitoring of glucose and ketone levels using a KetoMojo device, initially reporting readings via text and later through Bluetooth-enabled app syncing. Continuous actigraphy was deployed over a 9-week period to monitor rest/activity patterns, using 50 Hz AX3 actigraph devices worn in succession for three weeks each. GGIR software for Windows R Package (CRAN, the Netherlands; see https://cran.r-project.org/package=GGIR) was used for accelerometer data processing. Daily ecological momentary assessments (EMAs) captured changes in anxiety, mood, energy levels, impulsivity and speed of thought, initially via text messages and subsequently via an online tool.

#### Post-intervention evaluation

Following the intervention, semi-structured telephone interviews were conducted with 15 participants and four research clinicians involved in the study to facilitate a process evaluation, the findings of which are reported separately.^18^

#### Dietary intervention protocol

The intervention was a modified ketogenic diet, with a macronutrient distribution of 60–75% calories from fats and 5–7% from carbohydrates, with the remainder sourced from protein. In consideration of blood cholesterol and triglycerides, a preference for unsaturated fats was advised. Adjustments to individual macronutrient ratios were made throughout the study by the study dietitian based on various factors, including the attainment of ketosis defined by a target blood ketone level of 1–4 mmol/L and glucose levels of 4–7.8 mmol/L. In participants where weight loss was both desired and considered safe, a caloric deficit was prescribed to promote body fat as an alternative ketone source. Participants engaged in the diet for a 6–8 week duration, which included an initial adaptation phase, alongside continued standard medical care from their regular healthcare providers.

#### Dietary support and management

Throughout the intervention, participants had weekly remote consultations with a dietitian, supplemented by additional contacts as needed. Participants received guidance to ensure understanding of the diet's principles, support in adhering to the diet, assistance with troubleshooting dietary challenges and management of potential side-effects. The diet plans specified total caloric and macronutrient (in grams) daily intake, portioned accordingly. Customised recipes were provided to align with individual dietary needs. Supervision was continued throughout the diet's cessation phase, with support offered to those opting to maintain the dietary changes. Behavioural strategies were integrated to enhance diet adherence, including adherence checklists completed once every 2 weeks guided by the COM-B framework (which highlights the influence of capability, opportunity and motivation on behaviour). These have been analysed in a separate process evaluation.^18^

Excluding the baseline meeting, the median time the study dietitian spent with each participant was 505 min. This time was spent supporting adherence to the diet, confirming understanding of the intervention, problem solving and identifying and managing side-effects. The study dietitian kept a record of contact, which is summarised in our previous publication and the main points given briefly here.^14^ Thirty-two changes to the diet prescriptions were made throughout the study ranging, from zero to five per participant (median = 1). The ketogenic diet was designed to induce ketosis (as measured daily by participants) and consisted of the same macronutrient ratios for all participants at baseline. These were adapted where necessary to maintain ketosis in the participants, according to their individual responses to the diet. Personalised recipes were provided for each participant based on their individual calorie requirements and dietary preferences.

#### Nutritional supplementation and monitoring

Consistent with international nutritional guidelines, participants were counselled on increasing fluid intake and supplementing their diet with a broad-spectrum multivitamin and mineral supplement, along with a calcium and vitamin D supplement. Participants were also educated on managing possible adverse effects, such as hypoglycaemia or hyperketosis. Pre-intervention and follow-up health screenings, including analyses of urea and electrolytes, liver function tests and lipid profiles, were mandatory for all participants. These were carried out to rule out significant hepatic or renal dysfunction or familial hypercholesterolemia and to monitor for adverse changes related to diet, such as alterations in liver function or lipid levels.

#### Primary and secondary outcomes

The primary outcomes of this study assessing feasibility, acceptability and safety have been reported in a previous publication.^14^ Here we report the secondary outcome measures (ISRCTN61613198).

- Mood stability measured by the ALS-18 and depression by the BDI at baseline and at 8-week follow-up.
- Hypomania/mania symptoms measured using the YMRS at baseline and at 8-week follow-up.
- Glucose/ketone ratio measured using Ketomojo devices daily throughout the 10-week study period.
- Identification of specific metabolic changes in glucose, ketones and tricarboxylic acid (TCA) metabolites associated with the ketogenic diet, measured using serum and brain MRI measures at baseline and at week 8.
- Mood, energy, speed of thought, impulsivity and anxiety measured using visual analogue scales from 1 to 100 on daily EMAs during the 10-week study period.
- Episodes of bipolar depression and hypomania/mania occurring during the study period assessed by the MINI Neuropsychiatric assessment at baseline and at week 8.
- Sleep duration and circadian activity/rest rhythmicity parameters, assessed by actigraphs throughout the 10-week period (to include 2 weeks of stepping down the diet).

### Data management and statistical analysis

#### Clinical and metabolic outcomes

The statistical analysis used standard methods in SPSS and Excel software (Windows PC version). We calculated mean and median values, standard deviations and the change from baseline to follow-up for metabolic parameters, BDI, ALS-18 and YMRS values. We used the paired samples *t*-test function in SPSS to compare the mean at baseline and follow-up and determine statistical significance (defined as *P* < 0.05). Available case analysis of all participants who had baseline and follow-up measures was performed and the sample sizes for each variable are given in the results. Homeostatic model assessment of insulin resistance (HOMA-IR) was calculated from fasting insulin and glucose levels using the standard equation.^19^

#### Ecological momentary assessment and analysis

Text messages were used for daily EMA of mood, energy, speed of thought, impulsivity and anxiety. During the study, the initial EMA rating instructions delivered to participants 1–12 were highlighted by participants as difficult to follow because of inconsistent individual interpretations of normal and pathological ranges on the 100-point scale (e.g. in the mood category participants chose differing ranges between 1 and 100 to indicate their healthy mood, depression and hypomania, making comparisons unworkable). Thresholds for normal ratings and pathological ratings of depression and (hypo)mania were therefore added to the instructions for patients 13–27 (see Supplementary material). Note that EMA analyses were performed only on this second subgroup (*n* = 14).

#### Brain magnetic resonance imaging and spectroscopy

MRS data were processed using Osprey for Windows (MATLAB, Open Source, v2.4.0; see https://github.com/schorschinho/osprey)^20^ including frequency alignment and phasing of transients, co-registration to the T1w anatomical image and segmentation of tissues within the voxels. Data were fitted in the frequency domain using a linear combination modelling approach, including default basis functions for lipid, macromolecular resonances and metabolites (ascorbate, aspartate, creatine, gamma-aminobutyric acid, glycerophosphorylcholine (GPC), glutathione, glutamine (Gln), glutamate (Glu), myo-inositol, lactate, *N*-acetylaspartate (NAA), *N*-acetylaspartylglutamate (NAAG), phosphocholine (PCh), phosphocreatine (PCr), phosphoethanolamine, scyllo-inositol and taurine). Of the metabolites, tNAA (NAA + NAAG), Glx (Gln + Glu), tCr (PCr + creatine), tCho (PCh + GPC), myo-inositol, Glu and Gln were quantified with sufficient reliability for inclusion in further analysis and are reported here. Metabolite levels were quantified as water-scaled, relaxation-corrected and tissue-corrected estimates of molal concentration.^21^ Spectra were automatically excluded if either the creatine or water resonance linewidth (full width at half maximum) was equal to or greater than 0.1 ppm; in addition, spectra and model fits were inspected visually and excluded in case of poor model fit, excessive lipid contamination, spurious signal contamination, low signal, poor water suppression or baseline distortion.

#### Metabolomics

Fasting blood samples were taken at baseline appointments and at 6–8 week follow-up appointments. Global metabolomics analysis was performed on a total of 36 serum samples, corresponding to 18 participants who gave a baseline and follow-up blood sample and included one unfasted participant. Standard biochemical parameters included HbA1c, C-reactive protein (CRP), beta-hydroxybutyrate (BHB), insulin, glucose and lipid levels. A more detailed exploratory investigation of untargeted metabolomics was also performed using Rapid HILIC-Z Ion mobility mass spectrometry (RHIMMS) analysis to probe the serum metabolome, annotating a total of 358 metabolic features. Please see Supplementary materials for further details.

## Results

The primary outcomes for this study (acceptability and feasibility) have been published previously: 20 out of 26 participants who started the diet adhered to the intervention and completed the outcome assessments.^14^

### Clinical outcomes

All participants were clinically euthymic at baseline and median scores at week 8 remained within euthymic range, with no statistically significant changes: ALS: 15 (interquartile range (IQR) = 16.5) to 12.5 (IQR = 13.25) (*P* = 0.80) (*n* = 18); YMRS: 0 to 0 (*P* = 0.86) (*n* = 20), BDI: 8.5 (IQR = 11) to 9 (IQR = 9.25) (*P* = 0.55) (*n* = 18). The MINI Neuropsychiatric Interview assessments reported one episode of hypomania during the study period.

**Fig. 1:**
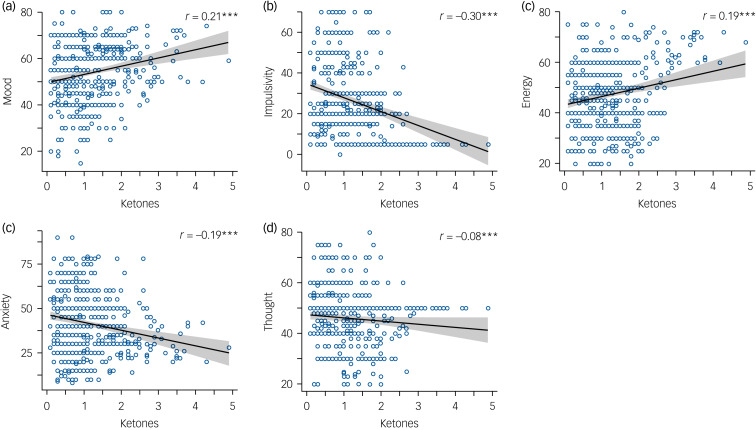
Daily ecologicalmomentary assessment and ketone levels (for a subset of *n* = 14 participants).

Daily EMA measurements of mood, energy, speed of thought, impulsivity and anxiety on a subset of participants who provided reliable EMA data (*n* = 14) were plotted against daily ketone levels (Fig. 1).

A positive correlation was observed between daily ketone level and mood (*r* = 0.21, *P* < 0.001) and energy (*r* = 0.19, *P* < 0.001) scores, with a negative correlation between ketone levels and both impulsivity (*r* = −0.30, *P* < 0.001) and anxiety (*r* = −0.19, *P* < 0.001). No correlation was observed with speed of thought (*r* = −0.08, *P* > 0.05) (Fig. 1). Seven of the 20 participants who completed the intervention requested not to participate in the dietary cessation period and opted to continue on a ketogenic diet.

### Metabolic outcomes

At baseline, 70.3% of participants were obese or overweight. Mean body weight decreased by 4.2 kg (from 81.2 ± 13.2 to 77.0 ± 11.7 kg, *P* < 0.001) (*n* = 18). Mean BMI decreased by 1.5 kg/m^2^ from 28.2 ± 4.2 to 26.7 ± 3.9 kg/m^2^ (*P* < 0.001) (*n* = 20). Mean systolic blood pressure decreased by 7.4 mmHg from 133.1 ± 16.2 to 125.7 ± 11.9 mmHg (*P* = 0.041) (*n* = 19).

The changes in mean values of the standard biochemical parameters (reported below as mean values at baseline then at follow-up) were not statistically significant: mean diastolic blood pressure from 81.5 ± 12.20 to 77.4 ± 7.7 mmHg (*P* = 0.162) (*n* = 19); fasting insulin from 75.3 ± 44.0 to 68.4 ± 49.0 pmol/L (*P* = 0.540) (*n* = 17); fasting glucose from 5.0 ± 0.6 to 4.7 ± 0.4 mmol/L (*P* = 0.12) (*n* = 17); HbA1c from 34.9 ± 2.7 to 33.9 ± 2.3 mmol/mol (*P* = 0.153) (*n* = 16); high-density lipoprotein (HDL) cholesterol from 1.6 ± 0.43 to 1.5 ± 0.51 mmol/L (*P* = 0.72) (*n* = 18); low-density lipoprotein (LDL) cholesterol from 3.0 ± 0.71 to 3.6 ± 1.4 mmol/L (*P* = 0.09) (*n* = 18); total cholesterol from 5.3 ± 0.9 to 5.8 ± 1.7 mmol/L (*P* = 0.21) (*n* = 18); triglycerides from 1.5 ± 0.8 to 1.6 ± 0.9 mmol/L (*P* = 0.98) (*n* = 16); and CRP from 2.4 ± 1.8 to 2.8 ± 3.6 mg/L (*P* = 0.738) (*n* = 15).

Each sample size above reflects the number of participants who had complete data for the respective parameter (variations in sample size across different metabolites are because of missing data, which occurred as a result of incomplete sample collection, processing issues or participants missing specific tests).

### Serum metabolomics

Here we report data on glucose, ketones and TCA cycle-related metabolites (those that were measurable with our approach) as pre-specified study outcomes.

An exploratory global metabolomics analysis (358 metabolic features annotated) was performed on a total of 36 serum samples, corresponding to 18 participants who gave a baseline and follow-up blood sample, including one baseline non-fasted participant. As expected with human metabolome studies, large variations were noted between the metabolic profiles of individual participants. However, multivariate statistical analysis of the baseline and follow-up sample groups showed a clear separation of the study groups in partial least squares discriminant analysis (PLS-DA), suggesting that reliable trends in participant metabolism could be observed within this data-set. For the metabolomics principal component analysis (PCA) score plot showing the serum metabolome at baseline and follow-up, please see Supplementary Fig. 1. For the PLS-DA score plot showing separation of the serum metabolome at baseline and follow-up, and for box plots of the metabolomics results, please see Supplementary Fig. 2.

In the analysis comparing levels of metabolites between baseline and follow-up, for ketone bodies we observed increases in β-hydroxybutyrate (from 37 563 ± 36 445 to 277 866 ± 191 798 (*P* = <0.001)), β-ketopentanoate (from 1548 ± 1367 to 2121 ± 2243 (*P* = 0.361)) and acetone (from 5277 ± 7234 to 10 776 ± 20 957 (*P* = 0.3)). There were small reductions in acetoacetate (from 27 679 ± 8906 to 20 520 ± 12 377 (*P* = 0.054)) and beta-hydroxypentanoate (from 782 ± 1316 to 34 ± 146 (*P* = 0.022)). There was a reduction in glucose (from 210 550 ± 188 492 to 170 700 ± 202 785 (*P* = 0.545)). For TCA cycle-related metabolites, we observed a decrease in lactate (from 171 216 ± 171 955 to 59 291 ± 79 476 (*P* = 0.017)) and Gln (from 531 128 ± 170 513 to 472 478 ± 151 317 (*P* = 0.283)), an increase in citrate (from 940 ± 1927 to 3535 ± 4196 *P* = 0.023) and isocitrate (from 2674 ± 7548 to 4079 ± 8837 (*P* = 0.611)) and no change in cis-aconitate (from 3520 ± 2989 to 3504 ± 3179 to (*P* = 0.998)).

### Brain metabolite levels

The MRS data below includes all usable scans from the 27 participants, including those who did not complete the dietary intervention or attend for follow-up (baseline *n* = 25, follow-up *n* = 19; including one baseline non-fasted scan). MRS data from the RDLPFC was additionally missing for *n* = 1 participant at both visits since this acquisition was included after the study had begun. Some (*n* = 6) participants did not attend the follow-up imaging visit. Overall, *n* = 25 ACC, *n* = 25 PCC and *n* = 24 RDLPFC session one scans and *n* = 19 ACC, *n* = 19 PCC and *n* = 18 RDLPFC session two scans were acquired.

Automatic quality assessment of the MRS scans resulted in the exclusion of MRS scans of the ACC with linewidth ≥0.1 ppm from the first (*n* = 3) and second (*n* = 1) scanning sessions. No RDLPFC or PCC scans were automatically excluded. Additional spectra were excluded following visual inspection (*n* = 5 visit 1, *n* = 6 visit 2), all in the RDLPFC. Creatine and water line widths for included spectra were 6.30 ± 1.80 and 6.55 ± 0.88 Hz, respectively. After automatic and manual exclusions, good fit was achieved in *n* = 22 ACC, *n* = 25 PCC and *n* = 19 RDLPFC session one scans and *n* = 18 ACC, *n* = 19 PCC and *n* = 12 RDLPFC session two scans, and were therefore useable in the descriptive analysis. In addition, *n* = 16 ACC, *n* = 19 PCC and *n* = 11 RDLPFC paired data-sets were useable for calculating paired *t*-tests.

Neurometabolite concentration estimates are reported in Table 1. Glx decreased from baseline in both the ACC (concentration change: −1.50 ± 2.40 [−2.78, −0.22] mM) and PCC (−2.14 ± 1.94 [−3.08, −1.21] mM). A small decrease in total choline was observed in both the ACC (−0.17 ± 0.20 [−0.28, −0.06] mM) and PCC (−0.12 ± 0.12 [−0.18, −0.06] mM) and a decrease in myo-inositol was observed in the PCC (−0.39 ± 0.69 [−0.72, −0.06] mM). No significant differences in any of the metabolites were observed in the RDLPFC.

**Table 1:** Mean metabolite concentrations estimated using magnetic resonance spectroscopy before and after ketogenic diet intervention

Brain region	Metabolite	Baseline (mean ± s.d.) [95% CI] (mM)	Change (mean ± s.d.) [95% CI] (mM)	*P*
ACC	tNAA	16.40 ± 0.98 [15.97, 16.84]	+0.21 ± 1.12 [−0.39, +0.80]	0.47
	Glx	12.91 ± 2.98 [11.59, 14.23]	−1.50 ± 2.40 [−2.78, −0.22]	0.025
	tCr	12.93 ± 1.20 [12.39, 13.46]	−0.52 ± 0.98 [−1.04, + 0.00]	0.050
	tCho	2.83 ± 0.35 [2.67, 2.98]	−0.17 ± 0.20 [−0.28, −0.06]	0.0044
	Myo-inositol	10.45 ± 1.66 [9.71, 11.18]	−0.08 ± 1.61 [−0.94, + 0.77]	0.84
PCC	tNAA	17.17 ± 0.81 [16.83, 17.50]	−0.28 ± 0.64 [−0.59, + 0.03]	0.071
	Glx	15.76 ± 1.97 [14.95, 16.57]	−2.14 ± 1.94 [−3.08, −1.21]	0.00014
	tCr	11.64 ± 0.68 [11.36, 11.92]	−0.33 ± 0.75 [−0.69, + 0.04]	0.075
	tCho	1.85 ± 0.19 [1.77, 1.93]	−0.12 ± 0.12 [−0.18, −0.06]	0.00034
	Myo-inositol	8.76 ± 1.00 [8.35, 9.17]	−0.39 ± 0.69 [−0.72, −0.06]	0.025
RDLPFC	tNAA	16.50 ± 0.91 [16.06, 16.94]	+0.18 ± 0.79 [−0.35, +0.72]	0.46
	Glx	12.45 ± 2.48 [11.25, 13.64]	−1.61 ± 2.74 [−3.45, + 0.24]	0.081
	tCr	11.32 ± 0.75 [10.96, 11.69]	−0.21 ± 0.71 [−0.69, + 0.26]	0.34
	tCho	2.23 ± 0.27 [2.10, 2.36]	−0.02 ± 0.17 [−0.13, + 0.10]	0.74
	Myo-inositol	8.72 ± 1.18 [8.15, 9.29]	+0.34 ± 0.77 [−0.18, +0.85]	0.18

*P*-values were calculated using paired *t*-tests.

ACC, anterior cingulate cortex; PCC,  posterior cingulate cortex; RDLPFC, right dorsolateral prefrontal cortex; tNAA, total *N*-acetylaspartate; Glx, glutamate + glutamine concentration; tCr, total creatine; tCho, total choline.

### Actigraphy

The actigraphy data had low completion rates with only 65% of the expected data returned for the 20 participants completing the study.^14^ The available data derived from actigraphy had substantial day-to-day variability both within and between subjects, precluding any meaningful assessment of the effect of the diet intervention on sleep and/or rest–activity rhythmicity.

## Discussion

Here we report pilot study findings on the short-term effect of a modified ketogenic diet on clinical, metabolic and brain MRS outcomes in euthymic participants with bipolar disorder. Overall, we found preliminary evidence that ketone levels in the blood may be correlated with positive changes in daily mood, energy, impulsivity and anxiety (but not speed of thought). Level of ketosis is considered to be important for seizure reduction in epilepsy, with higher levels correlated with seizure control.^22^

There was a positive effect of the ketogenic diet on some cardiometabolic parameters, with improvements in weight and systolic blood pressure, and with no statistically significant effect on lipid profile. These metabolic changes occurred while participants remained on their current medications. One third of the participants completing the study opted out of the dietary cessation period to remain on a ketogenic diet beyond the study period. Similar observations of voluntary, continued adherence post-study are documented in ketogenic diet studies in epilepsy.^23,24^

Conditions of metabolic dysfunction (insulin resistance, metabolic syndrome, type 2 diabetes and related conditions) are highly prevalent in people living with bipolar disorder.^10^ In addition, there are significant metabolic side-effects of several medications used to treat bipolar disorder.^25^ The improvements in body weight and blood pressure over the 6- to 8-week period, while adhering to existing medications, were therefore encouraging signs of metabolic improvement. The lack of significant change in lipid parameters is also of interest, as a potential detrimental effect on lipid profile has been considered a risk of adhering to a ketogenic diet.

An umbrella review of meta-analysis of clinical trials of ketogenic diet on health outcomes reports improvements in several cardiometabolic parameters, including body weight, HbA1c, insulin resistance, fat mass, triglycerides and diastolic blood pressure. However, the review also reported increased LDL.^26^ The question of whether elevations in LDL should be of concern in the context of a profile of improvement in other metabolic markers is a topic of ongoing scientific debate. A meta-analysis of 41 RCTs examining LDL cholesterol changes on a ketogenic diet indicates that people with high BMI typically experience no change or a reduction in LDL cholesterol and that elevations in LDL occur primarily in a subset of low BMI individuals.^27^ It has been suggested therefore that such elevations may represent an adaptation to increased mobilisation of fat as a metabolic fuel source, and increased reliance on lipid transport mechanisms, occurring primarily in lean individuals.^27^ However, this remains an area of scientific debate and in patients who experience significantly elevated LDL on a ketogenic diet, it is not yet clear whether this represents increased cardiovascular risk.^28^ This pilot study did not find significant adverse changes in lipid profile on average over the 6–8 week duration. However, this is clearly an issue of potential concern, and worthy of investigation in larger trials.

We observed preliminary evidence that the ketogenic diet was associated with decreased brain Glx in the ACC and decreased Glx and myo-inositol in the PCC. Elevated Glu is among the most consistent findings in brain MRS studies of bipolar disorder^29–31^ and Glu metabolism is proposed as a mechanism of action of several antiseizure medications shared between epilepsy and bipolar disorder. Our observation of decreased Glx post-intervention is in line with previous research detecting a reduction in Glu, Gln and Glx in people with bipolar disorder who were good responders to pharmacological interventions^32–36^ and to total sleep deprivation and light therapy.^37,38^ We have previously proposed possible roles of Glu and myo-inositol in cerebral metabolism and insulin signalling in bipolar disorder.^9,39^ These findings are preliminary from a small-scale study but merit further investigation in studies exploring potential biological mechanisms shared among bipolar disorder, antiseizure medications and ketosis.^39^

In the targeted metabolomics analyses of TCA-related metabolites, we observed a reduction in serum lactate. Elevated lactate is also a consistent serum biomarker in bipolar disorder indicated by systematic review.^40^ It has been proposed as an indicator of mitochondrial dysfunction in bipolar disorder and other neurological conditions that are accompanied by psychiatric symptoms.^41^ However, serum lactate is primarily muscular in origin and therefore may have limited or no correlation to brain lactate. While lactate may cross the blood brain barrier via monocarboxylate transporters,^42^ the net movement of lactate between blood and brain is limited and there is a relatively small volume and metabolic turnover of lactate in the brain. Therefore, our finding of reduced lactate is likely to represent a downregulation of glycolysis in muscle and peripheral tissue rather than a change in the brain or central nervous system. Future research could study brain lactate with MRS in response to a ketogenic diet and compare to it serum measurements to better understand the dynamics between peripheral and central lactate.

As a pilot study to inform the design of a future RCT, there are a number of important limitations. Importantly, this study was not powered to demonstrate statistically significant differences in mental health, metabolic or brain MRS outcomes, so many of the findings reported above are preliminary. The limited sample size also means less precise parameter estimates, increased risk of false positive and false negative findings. We have also not corrected for multiple testing. Further, all participants – many of whom were interested in nutritional approaches – were recruited from a bipolar disorder charity and may not therefore be representative of patients recruited from a clinical setting. The design of this study was single-arm with no control group and was unblinded (for both participants and research staff) and so results may be subject to biases. Dietary interventions, such as the ketogenic diet, are not possible to blind in a trial. We present these data as exploratory and requiring replication and further validation within larger future studies.

## Conclusions

In this pilot study of a ketogenic diet intervention in euthymic individuals with bipolar disorder, we observed some evidence of potential mental health and metabolic benefits. Given the substantial cardiometabolic risk associated with bipolar disorder and the urgent need for new adjunctive (and non-medication) treatment strategies, replication of these findings and a RCT are now warranted. Tentatively, our findings suggest that at least a proportion of patients with bipolar disorder may benefit clinically from an adjunctive metabolic treatment approach such as the ketogenic diet.

## Supporting information

Campbell et al. supplementary material 1 Campbell et al. supplementary material

Campbell et al. supplementary material 2 Campbell et al. supplementary material

Campbell et al. supplementary material 3 Campbell et al. supplementary material

## Data Availability

The data that support the findings of this study are not publicly available as explicit consent was not sought from participants.
