# Supplementary figures and images for: A pilot study of a ketogenic diet in bipolar disorder: clinical, metabolic and magnetic resonance spectroscopy findings
Source: BJPsych Open. 2025 Feb 25;11(2):e34. doi: 10.1192/bjo.2024.841 (PMC12001942; doi:10.1192/bjo.2024.841)

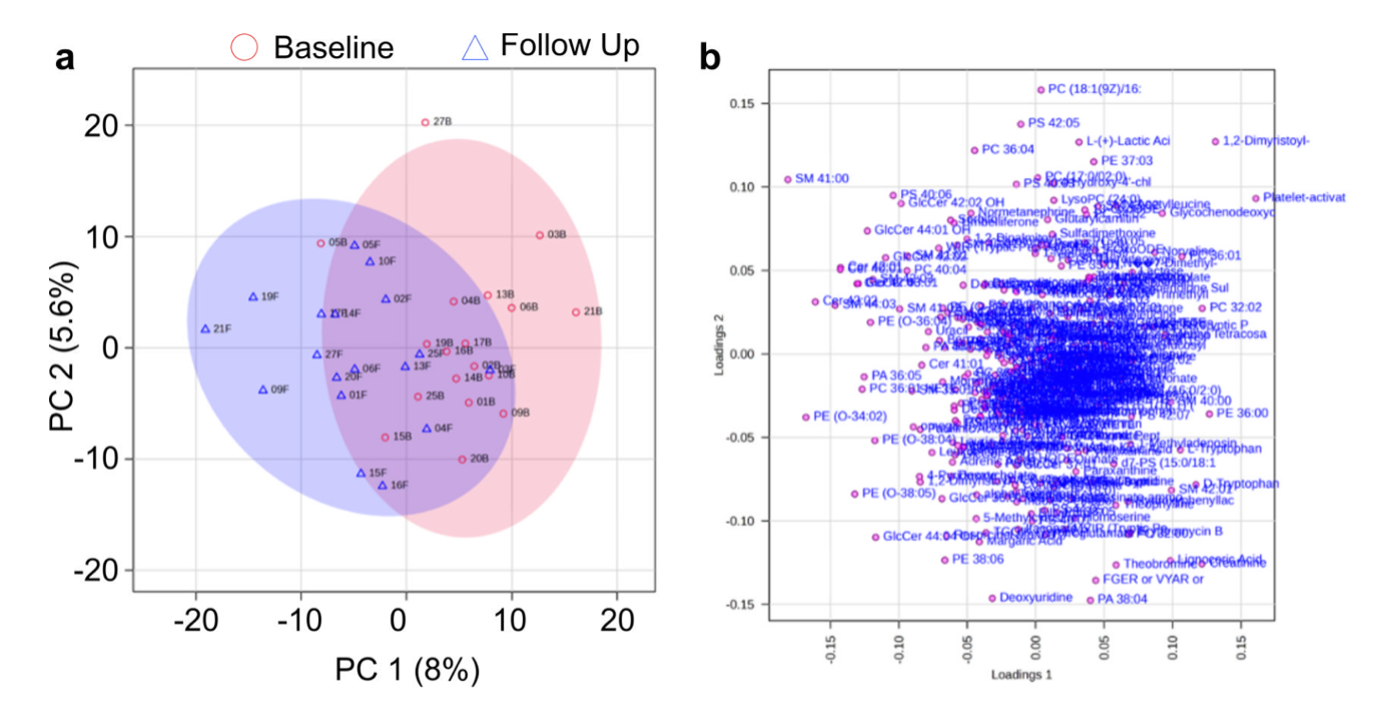

Supplement: Campbell et al. supplementary material 2 — Campbell et al. supplementary material [file S205647242400841Xsup002.png]

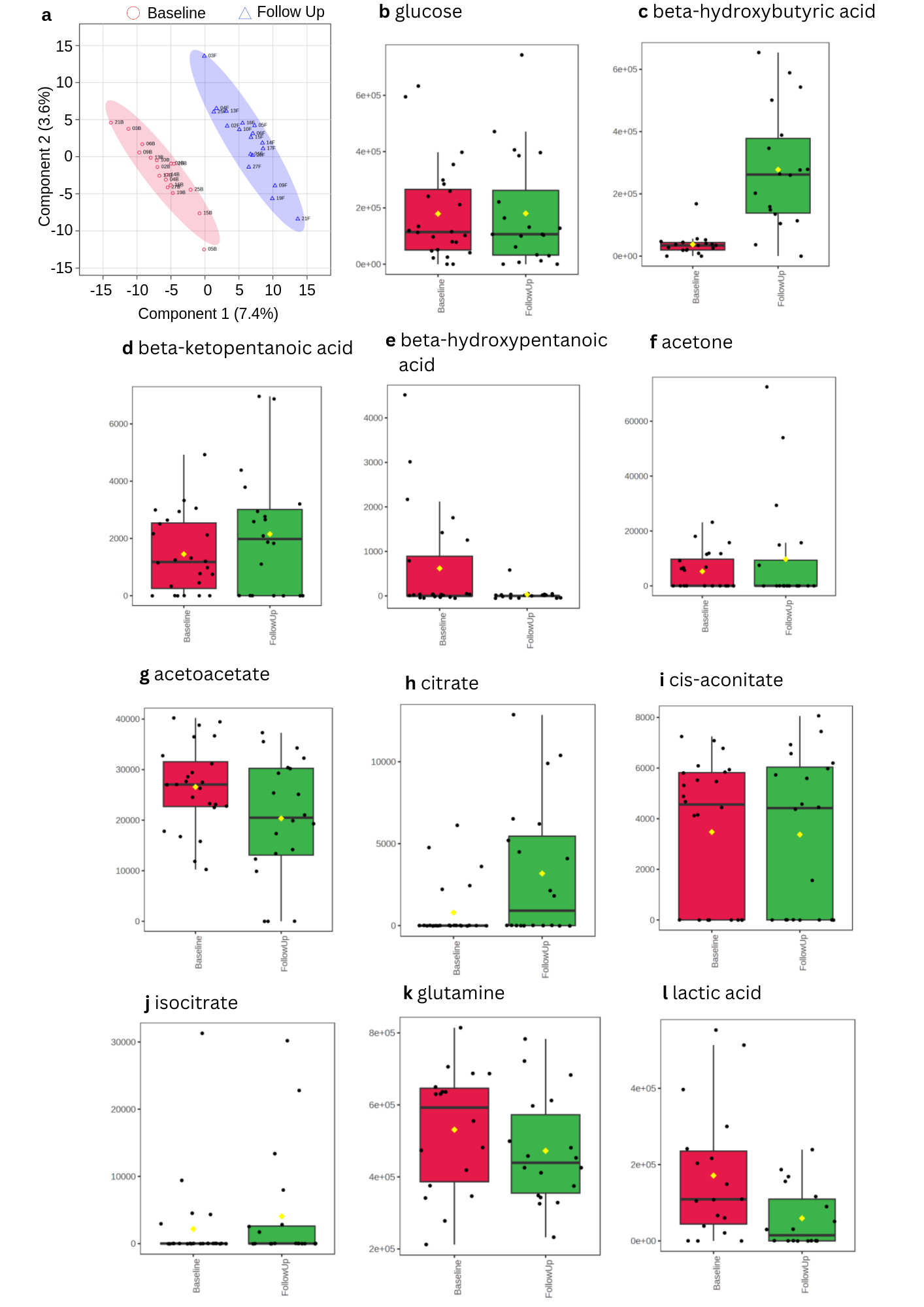

Supplement: Campbell et al. supplementary material 3 — Campbell et al. supplementary material [file S205647242400841Xsup003.png]
